# The Zn(II)2Cys6 transcription factor *Ds*Ace3 is a major activator of cellulases in the white-rot fungus *Dichomitus squalens*

**DOI:** 10.1128/aem.01548-25

**Published:** 2026-02-09

**Authors:** Victor M. Gonzalez Ramos, Joanna E. Kowalczyk, Henry N. Maina, Jayson Talag, Steven Ahrendt, Anna Lipzen, Jie Guo, Alicia Clum, Kurt LaButti, Hope Hundley, Kerrie Barry, Igor V. Grigoriev, Miia R. Mäkelä

**Affiliations:** 1 Department of Bioproducts and Biosystems, Aalto University 174277, Espoo, Finland; 2 Department of Microbiology, University of Helsinki 3835, Helsinki, Finland; 3 Department of Food and Nutrition, University of Helsinki 3835, Helsinki, Finland; 4 Arizona Genomics Institute 124686 , Tucson, Arizona, USA; 5 US Department of Energy Joint Genome Institute, Lawrence Berkeley National Laboratory 118576, Berkeley, California, USA; 6 Department of Plant and Microbial Biology, University of California Berkeley 1438, Berkeley, California, USA; Royal Botanic Gardens Kew, Surrey, United Kingdom

**Keywords:** *Dichomitus squalens*, CRISPR/Cas9, basidiomycetes, cellulose, transcription factor

## Abstract

Plant biomass degradation by fungal extracellular enzymes is tightly controlled at the transcriptional level, enabling fungi to respond to the complex and variable composition of plant cell wall. White-rot basidiomycetes are of special interest due to their unique ability to degrade all components of lignocellulose, yet information on their transcriptional regulators involved in this process is highly limited. In this study, we characterized *Ds*Ace3, a Zn(II)2Cys6 transcription factor from the white-rot fungus *Dichomitus squalens*, predicted to be orthologous to the cellulase activators Ace3 of the ascomycete fungus *Trichoderma reesei* and Roc1 of the basidiomycete fungus *Schizophyllum commune*. Disruption of *Ds*Ace3 suppressed extracellular cellulase production and significantly impaired growth on cellulose and cellobiose. Transcriptomic analyses also confirmed that *Ds*Ace3 is essential for activating not only cellulase- and xylanase-encoding genes but also sugar transporter-encoding genes, linking enzyme production to sugar sensing and uptake. *Ds*Ace3 shows a strong phylogenetic and functional conservation compared to its ascomycete counterpart in *T. reesei*, a rare feature in known regulators of lignocellulose degradation. However, differences in the response of *Ds*Ace3 to lactose indicate distinct substrate sensing or signaling mechanisms in basidiomycetes. This study describes the first functional characterization of an Ace3 ortholog in a strongly ligninolytic white-rot fungus and provides critical insights into transcriptional regulation in white-rot fungi, facilitating advances in fungal applications for biomass conversion.

**IMPORTANCE:**

White-rot basidiomycete fungi are key organisms in natural carbon cycling due to their unique ability to break down all polymeric components of wood. Despite their ecological and biotechnological importance, little is known about how these fungi control the genes responsible for this process. In this study, we characterized a transcription factor, *Ds*Ace3, in the white-rot fungus *Dichomitus squalens*. We show that *Ds*Ace3 is essential for activating genes needed to degrade cellulose and import the sugars released. Notably, *Ds*Ace3 shares functional conservation with similar regulators in both ascomycete and basidiomycete fungi. Our findings offer new insights into how wood-decaying white-rot fungi regulate plant biomass degradation and provide a molecular foundation for improving fungal enzyme production in industrial and environmental applications.

## INTRODUCTION

Plant biomass-degrading fungi play a crucial role in global carbon cycling by decomposing lignocellulosic plant biomass, a complex matrix of cellulose, hemicellulose, lignin, and minor polysaccharides (1). These fungi typically secrete a diverse array of carbohydrate-active enzymes (CAZymes), mostly from families of glycoside hydrolases (GHs), glycosyl transferases, polysaccharide lyases, carbohydrate esterases, and auxiliary activities (AAs) (2), to break down plant polymers into mono- and small oligomers, which fungi utilize as carbon and energy sources (3–5). Fungi from Ascomycota and Basidiomycota often exhibit conserved genomic potential for extracellular enzymatic degradation, as evidenced by the similar distribution of polysaccharide-related CAZy families across these phyla (6). However, the composition of biomass is highly variable across plant species, requiring that fungi adapt their molecular responses according to their substrate preference (3, 7).

Transcriptional regulation is a key mechanism enabling fungi to optimize enzyme production in response to their environment. Plant polysaccharide degradation in filamentous fungi is tightly controlled by transcription factors (TFs) that are induced by specific sugar compounds (8, 9) and their metabolic conversion products (10, 11). Additionally, these TFs repress the expression of plant polysaccharide-converting genes in the presence of readily metabolizable carbon sources via carbon catabolite repression (CCR) (12) and respond to environmental cues such as pH and light (13, 14). TFs from the Zn(II)2Cys6 and Cys2His2 families are prominent among these proteins, with the Zn(II)2Cys6 family being particularly widespread and functionally diverse, influencing the expression of genes involved in nutrient utilization (15, 16). The study of TFs enhances our understanding of how filamentous fungi sense and degrade plant biomass, providing insights into their evolutionary adaptation to various biotopes (17, 18) as well as genetic engineering strategies to develop existing or new production hosts for industrial processes (19, 20). Despite significant progress in identification and characterization of these regulators in ascomycetes, their functional counterparts in basidiomycetes remain understudied (15, 21).

Wood-degrading basidiomycetes, particularly white-rot fungi, are distinguished by their ability to degrade all components of lignocellulose, including the recalcitrant aromatic polymer lignin, highlighting the importance of investigating their molecular regulation (22–26). Recent (post-)genomic studies have identified candidate regulatory proteins in basidiomycetes, shedding light on their lignocellulose degradation strategies (27–30). However, beyond the general carbon catabolite repressor Cre1/CreA, which is conserved across Dikarya fungi, *Trichoderma reesei* Ace3 from the Zn(II)2Cys6 family is the only plant biomass degradation-related TF with predicted orthologs in both Ascomycota and Basidiomycota (15, 31).

In *T. reesei*, *Tr*Ace3 activates cellulase production, modulates xylanase (XLN) expression, and influences lactose metabolism, emphasizing its industrial relevance in the production of cellulolytic cocktails (31–34). Its predicted ortholog, *Sc*Roc1, in the basidiomycete *Schizophyllum commune* induces cellulases when cultivated on cellulose (15, 29). However, the extent to which these TFs and their orthologs are functionally conserved in ascomycetes and basidiomycetes is still unclear.

The white-rot basidiomycete *Dichomitus squalens* possesses a predicted ortholog of *Tr*Ace3 (15). It is also an interesting model organism for lignocellulose degradation due to its flexible physiology (22, 24) and ability to colonize both softwoods and hardwoods (35, 36). Our transcriptomic studies have shown that *D. squalens* induces cellulase genes in response to cellulose, with cellobiose acting as the primary inducer (28, 37). We also identified a cellulose-induced regulon that includes *Ds*Ace3, alongside genes encoding cellulose-degrading enzymes, predicted cellodextrin/lactose sugar transporters (STs), and a galactose metabolic enzyme, indicating functional links to *Tr*Ace3 and *Sc*Roc1 (28). Nevertheless, the molecular mechanisms underlying this induction and the specific regulatory role of *Ds*Ace3 remain unresolved.

In this study, we aimed to elucidate the regulatory function of *Ds*Ace3 in *D. squalens*, focusing on its control of cellulose degradation and cellobiose metabolism. By integrating phylogenetic, phenotypic, and transcriptomic analyses, we evaluated its role and functional conservation relative to *T. reesei Tr*Ace3 and *S. commune Sc*Roc1. This report on a characterized Ace3 ortholog in the strongly ligninolytic white-rot fungus *D. squalens* advances our understanding of transcriptional regulation in basidiomycetes and its relevance for lignocellulose conversion.

## RESULTS

### Updated genome sequence of *D. squalens* CBS 464.89

Here we present an updated version of the *D. squalens* CBS 464.89 genome (v.2.0) available at https://mycocosm.jgi.doe.gov/Dicsqu464_2, improving upon the previously published v.1.0 genome (38). A statistical summary comparing both assemblies can be found in Table S1. This update enables more accurate functional annotation, enhancing the resolution of analyses. Specifically, v.2.0 is a more contiguous assembly at 45.6 Mb in 60 scaffolds, compared to 39.6 Mb in 467 scaffolds of v.1.0. The improved assembly also captured longer repeat regions (15.51% vs 4.91%) dispersed throughout the genome. Additionally, 11 of 13 contigs >2 Mbp show telomere repeats (TTAGGG) on both termini, while the remaining 2 show them on only one terminus. The annotation yielded slightly fewer genes overall (13,877 vs 15,295), but a greater proportion of those genes were complete (97.18% vs 92.81%). Using BUSCO (v.5.0) with the fungi_odb10 lineage data set, we found that there were slightly fewer single-copy complete genes (97.23% vs 97.63%), along with more duplicated genes (2.11% vs 0.53%). The annotation of the improved assembly identified a fewer number of fragmented and missing genes (0.40% vs 1.06% and 0.26% vs 0.79%). Using Orthofinder, we evaluated gene conservation between the two versions. Ortholog groups with identical gene numbers were classified as conserved. Sequences of curated genes encoding putative CAZymes, TFs, STs, and sugar metabolic enzymes were manually reviewed to map Protein IDs to v.2.0 (Table S2). Unless stated otherwise, all identifiers in this study refer to *D. squalens* CBS 464.89 v.2.0 protein IDs.

### Ace3/Roc1 orthologs share conserved regulatory domains

To confirm the ortholog relationship of *Ds*Ace3 with *Tr*Ace3 and *Sc*Roc1, we performed a genome-wide analysis of ortholog clusters across 35 Dikarya fungi, including 18 ascomycete and 17 basidiomycete species. All identifiers refer to the protein IDs from the retrieved proteomes in Table S3. As expected, *Ds*Ace3, *Tr*Ace3, and *Sc*Roc1 clustered into a single ortholog group of 105 proteins. InterProScan analysis revealed that 28 of these proteins shared the PF00172 fungal Zn(II)2Cys6 binuclear cluster domain and the PF0482 fungal-specific TF domain, consistent with the domain architecture of *Ds*Ace3, *Tr*Ace3, and *Sc*Roc1.

**Fig 1:**
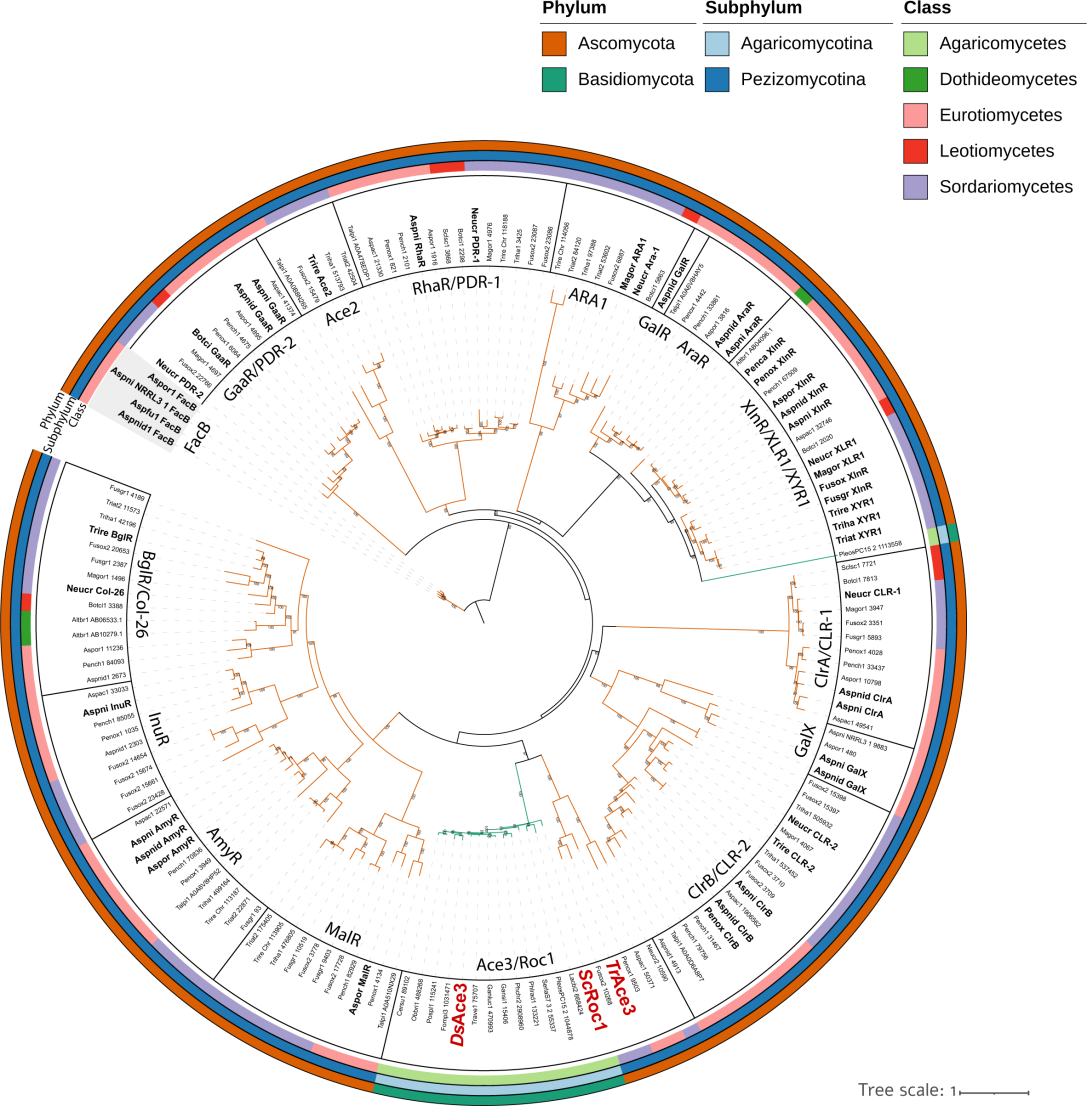
Pruned maximum-likelihood phylogenetic tree of selected Zn(II)2Cys6 fungal transcription factors involved in the regulation of plant biomass-modifying enzymes and their predicted orthologs from Ascomycota (orange) and Basidiomycota (green). Clades corresponding to distinct transcription factor groups (e.g., Ace3/Roc1, XlnR/XYR1, and AmyR) are indicated. Protein IDs from the source proteome and species abbreviations (as listed in Table S3) are shown. Experimentally characterized transcription factors are indicated in bold (as listed in Table S4). *Dichomitus squalens Ds*Ace3 and its characterized orthologs are highlighted in red and bold. Bootstrap support values ≥50 are shown at branch points.

Phylogenetic analysis included 720 protein sequences comprising the 28 identified proteins, 41 characterized Zn(II)2Cys6 TFs from eight additional ortholog groups associated with plant biomass utilization in filamentous ascomycetes, and proteins from these groups that matched the domain architecture of the characterized TFs (Table S4). The resulting tree placed *Ds*Ace3, *Tr*Ace3, and *Sc*Roc1 in a distinct clade, separate from other characterized TFs (Fig. 1; Fig. S1). This Ace3/Roc1 clade is further divided into two subclades: one containing 14 putative Ace3 proteins from basidiomycetes, including *Ds*Ace3 and *Sc*Roc1, and the other with *Tr*Ace3 and putative Ace3 proteins from the ascomycetes *Aspergillus aculeatus*, *Fusarium oxysporum*, *Neurospora crassa*, and *Penicillium oxalicum*. This grouping reflects the evolutionary divergence between ascomycetes and basidiomycetes.

**Fig 2:**
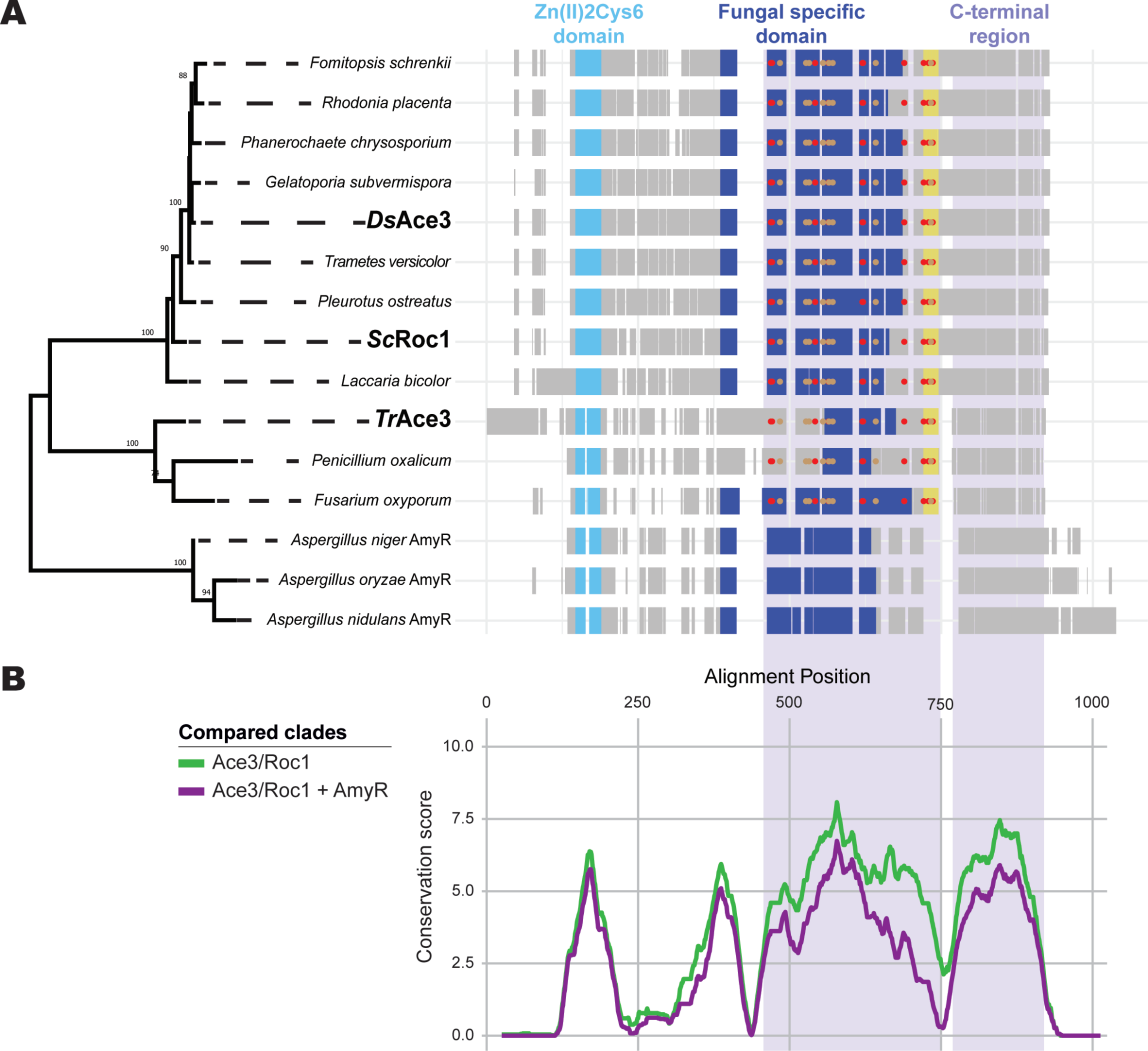
Sequence alignment and phylogenetic analysis of selected Ace3/Roc1 orthologs and characterized AmyR transcription factors from Ascomycota and Basidiomycota. (**A**) Maximum-likelihood (ML) phylogenetic analysis performed in MEGA11 using partial deletion of gaps (>95%) and the Poisson-corrected substitution rates. Bootstrap support values (500 resamplings) ≥50 are shown at branch points. Experimentally characterized Ace3/Roc1 orthologs are indicated in bold. A schematic representation of the ClustalW-aligned (https://www.ebi.ac.uk/jdispatcher/msa/clustalo) protein sequences and transcription factor domains is shown alongside the ML tree. Hydrophobic and flexibility-related residues conserved in Ace3/Roc1 are indicated by red and brown dots, respectively. Ace3/Roc1-specific region is highlighted in yellow. (**B**) Smoothed (*k* = 20) per-residue AMAS conservation scores of aligned transcription factors. Regions showing increased conservation within clades are highlighted in light purple.

Detailed sequence alignment of *Ds*Ace3, *Tr*Ace3, and *Sc*Roc1 with nine additional Ace3/Roc1 members and three AmyR TFs from Aspergilli revealed key conserved and divergent regions in these proteins (Fig. S2). The AmyR clade was selected due to its close phylogenetic relationship with Ace3/Roc1 and the availability of characterized members. The Zn(II)2Cys6 binuclear cluster domain (PF00172) exhibited exceptionally high conservation across all 12 Ace3/Roc1 and three AmyR proteins supporting shared DNA recognition capabilities for motifs like CGG triplets (Fig. 2). In contrast, the fungal-specific TF domain (PF04082) and the C-terminal region showed strong conservation within the Ace3/Roc1 family but diverged from AmyR sequences. These patterns highlight a conserved core DNA-binding function across the proteins, with regulatory divergence likely tailoring Ace3/Roc1 and AmyR TFs to different inducing conditions or activation strategies reflecting distinct activation mechanisms proposed for these regions (39–42).

Several key amino acids within these domains stand out for their conservation and potential functional significance (Fig. S2). In the Zn(II)2Cys6 domain, the six cysteine residues are perfectly conserved, forming the zinc-binding motif essential for DNA interaction. Within the fungal-specific TF domain (PF04082), hydrophobic residues such as phenylalanine (F) at positions 469, 542, and 564, leucine (L) at 471, 621, and 686, and isoleucine (I) at 620, 626, and 689, alongside flexibility-related amino acids like proline (P) at 484, 489, 532, and 642, alanine (A) at 527 and 571, and glycine (G) at 555, 565, 584, and 671, are highly conserved in Ace3/Roc1 TFs. These residues likely anchor a hydrophobic core or facilitate conformational flexibility. However, they differ in AmyR sequences, where gaps or substitutions predominate, supporting regulatory divergence between Ace3/Roc1 and AmyR proteins. Additionally, a unique region exclusive to Ace3/Roc1 immediately following PF04082 contains three conserved leucines and a proline, potentially enhancing structural stability or regulatory specificity in this clade.

### *Dsace3* disruption impairs growth and production of cellulases on cellobiose and cellulose

**Fig 3:**
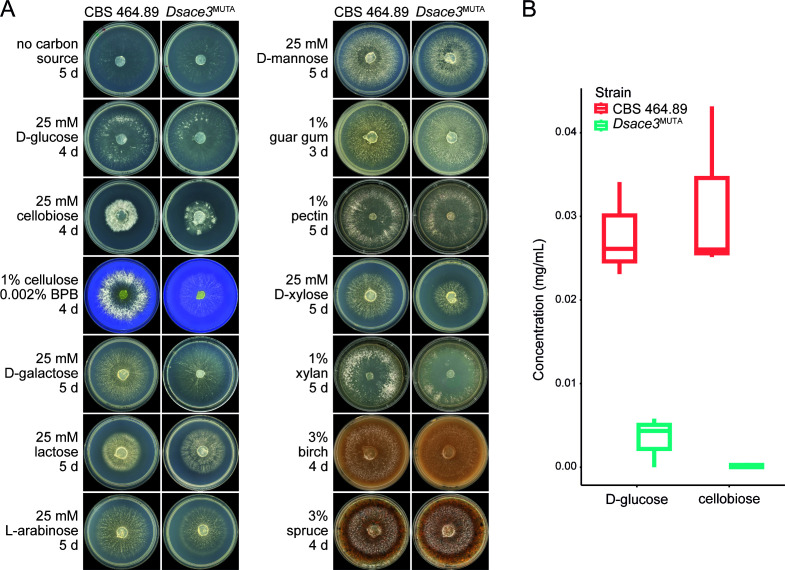
Phenotypic analysis of *ace3* disruption in *Dichomitus squalens*. (**A**) Growth profiling of the wild-type strain CBS 464.89 and *Dsace3*^MUTA^ strain on plant biomass-derived mono-, di-, and polysaccharides, wood substrates, and lactose. BPB, bromophenol blue. (**B**) Box plot of the high-performance anion-exchange chromatography with pulsed amperometric detection quantification of D-glucose and cellobiose in culture supernatants from triplicate cultivations of CBS 464.89 and *Dsace3*^MUTA^ grown on cellulose for 5 days.

To investigate the function of Ace3 in *D. squalens*, we generated three independent disruption mutant strains*—D. squalens ace3*^MUTA^, *ace3*^MUTB^, and *ace3*^MUTC^—using a CRISPR/Cas9 methodology (43) (Fig. S3A and B). The phenotypes of these mutants and the wild-type strain CBS 464.89 were compared after 3–5 days of cultivation on malt extract, glucose, cellobiose, cellulose, spruce, and birch. On cellobiose and cellulose, the mutants exhibited markedly reduced hyphal growth and thickness compared to the wild type (Fig. S3C). Based on these results, we chose *D. squalens ace3*^MUTA^ for further studies and performed extended growth profiling on additional plant biomass-derived mono-, di-, and polysaccharides, as well as the disaccharide lactose (Fig. 3A). As a result, minor reductions in mycelial density or diameter were also observed for *Dsace3*^MUTA^ on galactose, lactose, arabinose, xylose, and xylan.

To confirm that the observed phenotype on cellulose results from defective cellulose utilization, both *D. squalens ace3*^MUTA^ and the wild-type strain were pre-cultivated on malt extract for 3 days to achieve comparable mycelial growth and then transferred to liquid medium containing 1% cellulose for 2 days. The high-performance anion-exchange chromatography with pulsed amperometric detection (HPAEC-PAD) revealed no detectable levels of glucose or cellobiose (<0.005 mg/mL) in the *Dsace3*^MUTA^ culture liquids, whereas the wild-type cultivations resulted in 0.028 ± 0.01 mg/mL of glucose and 0.031 ± 0.01 mg/mL of cellobiose (Fig. 3B). This suggests that *Dsace3* disruption largely prevents the efficient extracellular degradation of cellulose in *D. squalens*.

**Fig 4:**
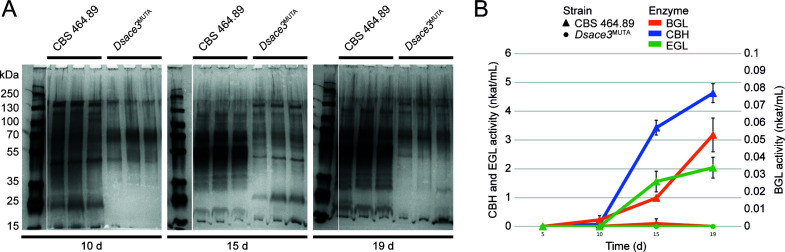
Effect of *ace3* disruption on extracellular protein production in *Dichomitus squalens*. Extracellular proteins and cellulase activity were analyzed from biological triplicate shaken liquid cultivations of the wild-type strain CBS 464.89 and *Dsace3*^MUTA^ strain grown on 1% cellulose. (**A**) SDS-PAGE analysis of extracellular proteins. (**B**) Cellulase activity. Error bars represent standard deviations of triplicate cultivations. BGL, β-glucosidase; CBH, cellobiohydrolase; EGL, endoglucanase.

Extracellular protein production by *Dsace3*^MUTA^ was analyzed in shaken liquid cultures containing 1% cellulose (Fig. 4) or 25 mM lactose (Fig. S4) and compared to the wild type. On cellulose, the mutant exhibited significantly reduced extracellular protein levels over time. Consistently, enzymatic activities for β-glucosidase (BGL), cellobiohydrolase (CBH), and endoglucanase (EGL) were undetectable in *Dsace3*^MUTA^, while the wild type displayed increasing activities over the cultivation period. These results demonstrate that *Ds*Ace3 regulates cellulose degradation in *D. squalens* by inducing the production of extracellular cellulases.

When cultivated on lactose, both strains resulted in comparable extracellular protein profiles with no variation over time (Fig. S4). Neither the wild-type strain nor *Dsace3*^MUTA^ showed enzymatic activities for any of the cellulases on lactose, suggesting that lactose does not support the induction of cellulases in *D. squalens*.

### Transcriptional activation of cellulases is highly dependent on *Ds*Ace3

Transcriptome analysis was conducted on *Dsace3*^MUTA^ and the wild-type strain to evaluate the molecular effects of the *ace3* disruption. Biological triplicates of both strains were initially grown on malt extract agar (MEA) for 3 days before being transferred to minimal agar medium supplemented with glucose, cellulose, xylan, or lactose for 2 days or grown directly on cellobiose for 5 days. To minimize noise, genes with low read counts (DESeq2 normalized counts <10 across all samples) were excluded. In total, 2,870 differentially expressed genes were identified using transcriptomes from D-glucose as a reference, a false discovery rate (FDR) of <0.05, and a fold-change (FC) threshold of ≥2.5, following criteria established in our previous study (Table S5) (28).

Principal component analysis showed that the first two principal components accounted for 52% of the total variance (Fig. S5). Samples clustered distinctly by substrate, with strain-specific separation observed on cellobiose and cellulose. The resulting divergence between wild-type and *Dsace3*^MUTA^ strains on cellulose-related substrates supports a regulatory role for *Ds*Ace3 in transcriptional responses to cellobiose and cellulose. In contrast, similar gene expression patterns were observed between the strains on glucose, lactose, and xylan, indicating that these pathways are not significantly influenced by *Ds*Ace3.

**Fig 5:**
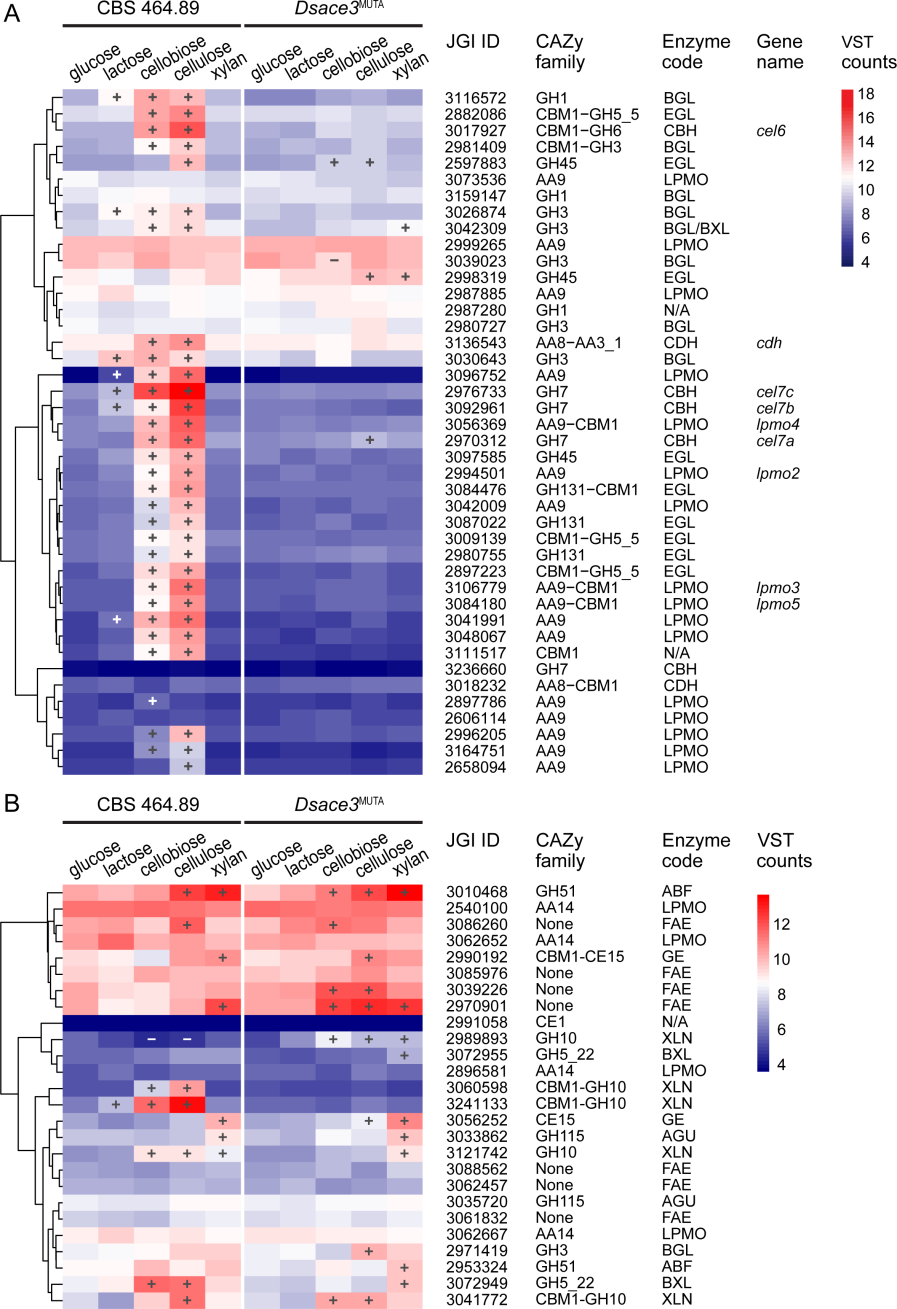
Hierarchical clustering of putative (**A**) cellulose-active and (**B**) xylan-active CAZy genes and feruloyl esterases (FAEs) from 5-day cultivations of *Dichomitus squalens* CBS 464.89 and *Dsace3*^MUTA^. Colors represent the mean variance-stabilized transformed (VST) expression values from biological triplicates. Significantly up- and downregulated genes are indicated by plus (+) and minus (−) symbols, respectively. Clustering was performed using complete linkage and Euclidean distance based on mean VST counts. JGI protein IDs correspond to the *D. squalens* CBS 464.89 v2.0 genome annotation available at the JGI MycoCosm database. Enzyme codes are listed in Table S2.

In the wild-type strain, cellobiose and cellulose strongly induced the expression of major cellulase-encoding genes, including *cel6*, *cel7a*, *cel7b*, *cel7c*, *cdh*, and most BGL and EGL encoding genes (FDR <0.05, FC ≥2.5) (Fig. 5A). Additionally, 12 of the 16 AA9 family LPMO-encoding genes were upregulated on cellobiose and/or cellulose. Consistent with the lack of cellulase activity observed in *Dsace3*^MUTA^, the mutant exhibited minimal cellulase induction, with only one GH45 *egl* gene upregulated on cellobiose, and two GH45 *egl* genes and one GH7 *cbh* gene (*cel7a*) induced on cellulose.

Together with cellulases, four of the five predicted GH10 XLN-encoding genes and two of the three predicted β-xylosidase (BXL)-encoding genes were induced in the wild-type strain on cellobiose and/or cellulose (Fig. 5B). However, only a single *xln* gene (Dicsqu464_2_3121742) was induced in the wild-type on xylan. In *Dsace3*^MUTA^, none of these *xln* genes were induced on cellobiose and cellulose, except for Dicsqu464_2_3041772. Interestingly, *xln* gene Dicsqu464_2_2989893, which was downregulated in the wild-type on cellobiose and cellulose, was upregulated in *Dsace3*^MUTA^. These results suggest that *Ds*Ace3 regulates the expression of genes encoding specific XLNs and BXLs in response to cellobiose but not to xylan.

Although lactose does not occur naturally in the ecological niches of *D. squalens* or *T. reesei*, it is commonly used as an inducer of cellulase production in industrial applications of *T. reesei*, where *Tr*Ace3 is essential for this process (31, 34). To assess whether a similar mechanism exists in *D. squalens*, we analyzed the transcriptomic profiles of lactose-supplemented cultures. In *Dsace3*^MUTA^, lactose did not induce any cellulase genes, while the wild-type strain exhibited modest induction of *cel7b* and *cel7c*, two AA9 *lpmos*, and one GH1 and two GH3 *bgl* genes. Additionally, none of the genes encoding three predicted GH35 β-galactosidases (Dicsqu464_2_3087851, Dicsqu464_2_3087970, and Dicsqu464_2_3087992) was induced by lactose (Table S5), indicating that *D. squalens* preferentially induces cellulases in response to cellobiose, a natural inducer for this species, rather than lactose.

To study if the disruption of *ace3* caused broader effects on the secretion machinery of *Dsace3*^MUTA^, we analyzed the expression levels of genes encoding oxidative enzymes related to lignin degradation and predicted extracellular peptidases. Additionally, we evaluated the transcription levels of the genes encoding predicted homologs of the Sec61 complex, which is an essential component of secretion machinery in eukaryotes (44). The expression levels of most lignin-degrading genes were comparable between mutant and wild type across all substrates, with the exception of four copper radical oxidases and three manganese peroxidases induced on cellobiose only in the mutant (Table S5). Predicted extracellular peptidases with signal peptides showed largely similar expression in both strains (Table S5). Similarly, all three genes encoding predicted orthologs of the Sec61 complex, i.e., Sec61 (Dicsqu464_2_3118201), Sbh1 (Dicsqu464_2_3011548), and Ss1 (Dicsqu464_2_2884003), showed constitutive expression in all conditions tested. Together with the phenotype of the three independent mutants, these results suggest that the transcriptional changes in cellulose expression observed in *Dsace3*^MUTA^ are an effect of targeted regulation and not a general disruption of the protein secretion machinery. However, protein-level assays of housekeeping secreted proteins would further strengthen this conclusion.

### *Ds*Ace3 partially regulates sugar transport and metabolism in *D. squalens*

To evaluate whether the *Dsace3* disruption affects plant biomass utilization beyond the regulation of CAZy genes in *D. squalens*, we analyzed the expression of predicted TF, ST, and sugar metabolic genes. The classification of STs and the association of sugar metabolic genes with sugar metabolic pathways were previously determined through orthology and phylogenetic analysis of characterized ascomycete proteins (45, 46). ST classifications are based on predicted preferred substrate—such as hexoses, pentoses, sugar acids, or cellodextrins/lactose—determined by homology to functionally characterized transporters. Similarly, sugar metabolic gene functions were inferred from their predicted enzymatic activity based on orthologs characterized within the sugar metabolism model of the ascomycete *Aspergillus niger* (45).

When cultivated on cellobiose and cellulose, *Dsace3* was induced 2.5- and 3.0-fold, respectively, in the wild-type strain but not in the *Dsace3*^MUTA^ (Table S5), which may suggest that *Ds*Ace3 is self-regulating similarly as has been observed in *T. reesei* and *S. commune* (29, 34). Additionally, the carbon catabolite repressor-encoding gene *Dscre1* (47) was induced only in the wild-type strain on cellulose. However, despite the induction of *Dscre1*, cellulose- and xylan-active CAZymes remained expressed, indicating a more complex interaction between CCR and CAZyme induction by *Ds*Ace3.

**Fig 6:**
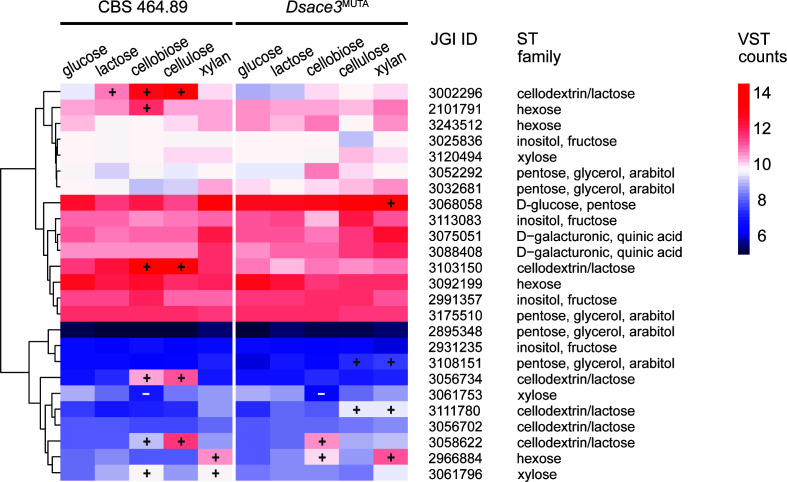
Hierarchical clustering of putative sugar transporter-encoding genes from 5-day cultivations of *Dichomitus squalens* CBS 464.89 and *Dsace3*^MUTA^. Colors represent the mean variance-stabilized transformed (VST) expression values from biological triplicates. Significantly up- and downregulated genes are indicated by plus (+) and minus (−) symbols, respectively. Clustering was performed using complete linkage and Euclidean distance based on mean VST counts. JGI protein IDs correspond to the *D. squalens* CBS 464.89 v.2.0 genome annotation available at the JGI MycoCosm database. Enzyme codes are listed in Table S2.

Our results indicate that sugar transport is partially regulated by *Ds*Ace3. Three of the six genes predicted to encode cellodextrin/lactose transporters (Dicsqu464_2_3002296, Dicsqu464_2_3056734, and Dicsqu464_2_ 3103150) were induced in the presence of cellobiose and cellulose in the wild-type strain but not in *Dsace3*^MUTA^ (Fig. 6), indicating that *Ds*Ace3 plays a role in controlling cellobiose uptake. However, one gene encoding these transporters (Dicsqu464_2_3103150) appears to be constitutively expressed, as it exhibited higher expression levels than the remaining STs across all substrates in both strains. Additionally, Dicsqu464_2_3002296 was also upregulated on lactose in the wild-type but not in *Dsace3*^MUTA^, indicating a reduced *Ds*Ace3-mediated response to lactose compared to cellobiose, similar to that observed for CAZyme expression.

**Fig 7:**
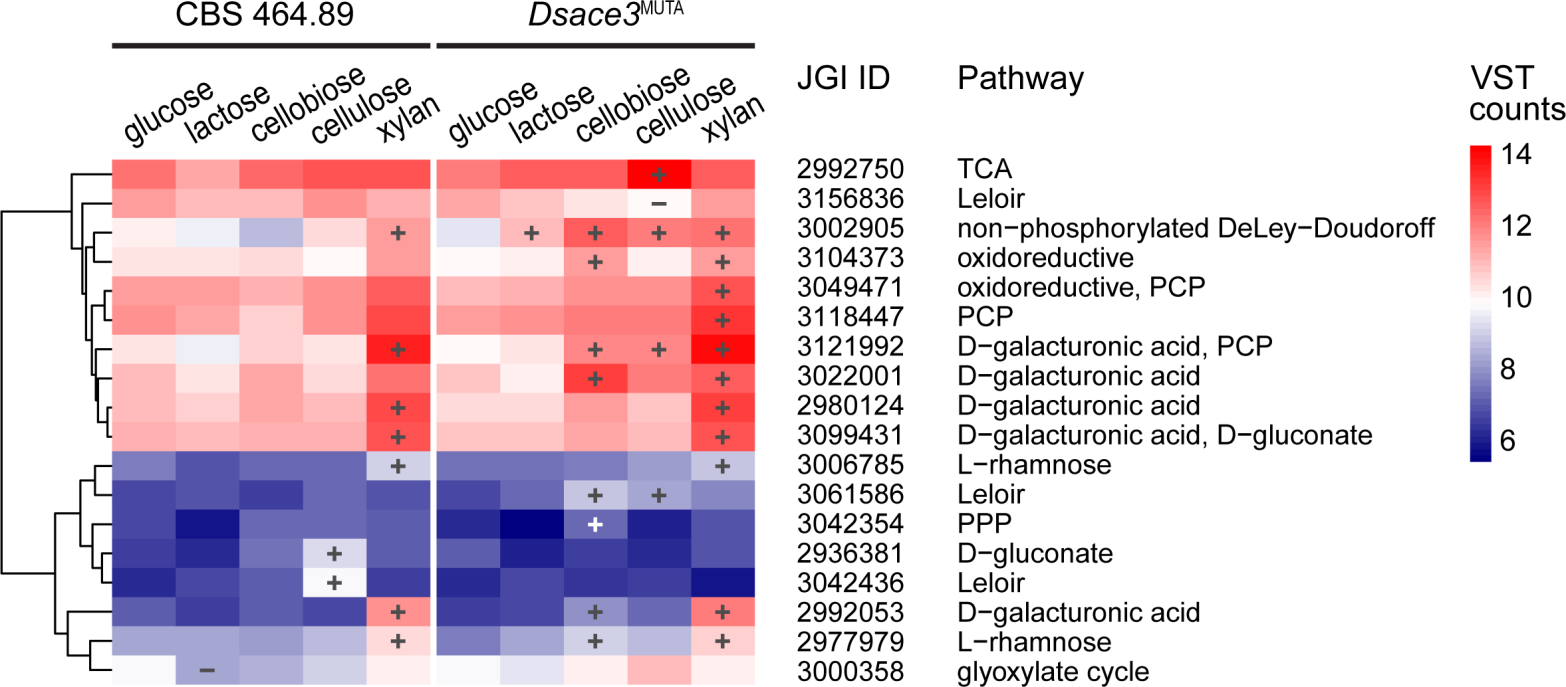
Hierarchical clustering of differentially expressed putative sugar metabolic genes from 5-day cultivations of *Dichomitus squalens* CBS 464.89 and *Dsace3*^MUTA^. Colors represent the mean variance-stabilized transformed (VST) expression values from biological triplicates. Significantly up- and downregulated genes are indicated by plus (+) and minus (−) symbols, respectively. Clustering was performed using complete linkage and Euclidean distance based on mean VST counts. JGI protein IDs correspond to the *D. squalens* CBS 464.89 v.2.0 genome annotation available at the JGI MycoCosm database. Enzyme codes are listed in Table S2.

Predicted sugar metabolic genes associated with galactose metabolism (Leloir, oxidoreductive, and non-phosphorylated DeLey-Doudoroff pathways), pentose metabolism (pentose catabolic pathway), galacturonic acid metabolism, and rhamnose metabolism (45) were induced in *Dsace3*^MUTA^ but not in the wild-type when cultivated on cellobiose and cellulose (Fig. 7). In contrast, a similar set of sugar metabolic genes was induced in both strains on xylan. Although this pattern could suggest that *Ds*Ace3 acts as a repressor, we hypothesize that the upregulation of these metabolic pathways in *Dsace3*^MUTA^ is a compensatory response in the mutant, as it is unable to efficiently metabolize cellobiose and cellulose.

When cultivated on cellulose, the wild-type strain showed induced expression of a gene encoding aldose-1-epimerase (Dicsqu464_2_3042436), which is predicted to function in galactose metabolism via the Leloir pathway (45). This induction was absent in *Dsace3*^MUTA^. Additionally, a gene encoding predicted UDP-glucose 4-epimerase (Dicsqu464_23156836), also involved in the Leloir pathway, was specifically downregulated in *Dsace3*^MUTA^ on cellulose but not in the wild-type strain. Notably, the expression levels of the genes encoding Dicsqu464_2_3042436 and Dicsqu464_2_3156836 did not show significant differences on any other substrate, including cellobiose, suggesting that *Ds*Ace3 may indirectly regulate this part of galactose metabolism specifically in response to cellulose degradation, possibly under low concentrations of the inducer cellobiose.

## DISCUSSION

Filamentous fungi tailor their transcriptional responses to efficiently utilize the diverse components of plant biomass. In ascomycete model organisms such as *T. reesei*, *Aspergillus* spp., and *N. crassa*, extensive studies have characterized TFs that regulate the expression of plant biomass-degrading CAZyme, ST, and sugar metabolic genes (21). In contrast, the regulatory mechanisms of basidiomycetes remain poorly understood, with only a few TFs functionally characterized to date (29). In this study, we addressed this knowledge gap by characterizing *Ds*Ace3, a Zn(II)2Cys6 family TF from the wood-degrading white-rot basidiomycete *D. squalens*, predicted to be an ortholog of *T. reesei* Ace3 and *S. commune* Roc1.

Phylogenetic analysis of the Zn(II)2Cys6 family revealed a distinct Ace3/Roc1 clade that includes (candidate) TFs from both ascomycetes and basidiomycetes and is separated from other characterized TF groups within the family. This Ace3/Roc1 clade is consistent with earlier observations by Benocci et al. (15), supporting the orthology between these TFs. In contrast, a previous study proposed a phylogenetic divergence between ascomycete and basidiomycete members of the Ace3/Roc1 clade based on HMM analysis describing *Sc*Roc1 as a non-ortholog of *Tr*Ace3 (29). While both studies rely on sequence homology and ortholog predictions, the diversity of roles played by orthologs—even within closely related fungal families—underscores the complexity of transcriptional regulation in plant biomass degradation (17, 18, 48).

A closer inspection of the sequence alignment and secondary structure analysis of Ace3/Roc1 clade members against three characterized AmyR TFs revealed higher divergence in the fungal-specific TF domain (PF04082) and C-terminal region between the clades, but greater conservation within the clades. While the role of PF04082 remains unclear, it has been hypothesized to be involved in TF regulation or effector recognition (39–41). These conserved structural features likely contribute to the shared regulatory mechanisms among Ace3/Roc1 orthologs.

Our results demonstrate that *Ds*Ace3 is essential for the induction of cellulose-active CAZymes in *D. squalens*. This function mirrors that of *Tr*Ace3 in the ascomycete *T. reesei*, a highly efficient cellulose degrader, and *Sc*Roc1 in the basidiomycete *S. commune*, which exhibits more limited ability to degrade wood (29, 31). We showed that the disruption of *Ds*Ace3 abolished cellulase production, leading to impaired growth on cellobiose and cellulose. Comparable phenotypes have been reported for the *T. reesei* ∆*Trace3* mutant, which displayed minimal cellulase activity, and for the *S. commune* ∆*Scroc1* mutant, which exhibited growth defects on cellulose and cellobiose (29, 31). While limited genetic tools prevented complementation of *Dsace3* in our study, previous work in *T. reesei* and *S. commune* suggests that restoring *Ds*Ace3 function would likely recover cellulase expression.

Cellobiose is the primary inducer of cellulase expression in *D. squalens* (28, 37), suggesting that sugar sensing and transport mechanisms are critical for cellulose metabolism. In *Dsace3*^MUTA^, the induction of predicted cellodextrin/lactose transporters was abolished, indicating that *Ds*Ace3 not only activates CAZyme expression but also regulates sugar uptake. A similar regulatory model has been described in *N. crassa*, where the CLR-1 and CLR-2 TFs activate CDT-1 and CDT-2 cellobiose transporters (8, 49–51). In *T. reesei*, *Tr*Ace3 regulates Crt1, a critical ST for cellulose sensing and utilization (52, 53). Similarly, in *S. commune*, several predicted STs were induced in the wild-type but not in the ∆*Scroc1* mutant (29). Although function prediction of fungal STs remains challenging (46, 54), our findings suggest a conserved role for Ace3/Roc1 orthologs in regulating CAZyme expression and sugar uptake. Further studies are needed to confirm the substrate specificity and regulatory function of individual transporters.

In addition to cellulase genes, *Ds*Ace3 also modulates the expression of xylan-degrading enzymes. Several predicted XLN and BXL encoding genes were induced in the wild-type on cellobiose and cellulose but not in *Dsace3*^MUTA^. A similar regulatory pattern is seen in *T. reesei*, where *Tr*Ace3 regulates xylanase expression both directly and indirectly through Xyr1, a major regulator of cellulase and xylanase genes (31, 34). In *S. commune*, deletion of *Scroc1* also led to reduced xylanase gene expression and impaired growth on xylan (29). However, the lack of a predicted Xyr1 ortholog and the limited induction of other TFs in *D. squalens* suggest that distinct regulatory mechanisms control xylanase induction in basidiomycetes compared to ascomycetes.

*Ds*Ace3 also influences lactose-related metabolism on cellulose, as indicated by the *Dsace3*^MUTA^ phenotype and loss of induction of an aldose 1-epimerase-encoding gene in the Leloir pathway (45). This resembles observations in *T. reesei* ∆*Trace3* (34), but the predicted β-galactosidase *bga1* and aldose reductase *xyl1* orthologs in *D. squalens* were unaffected, pointing to species-specific differences. While lactose drives cellulase induction in *T. reesei* via *Tr*Ace3 and Crt1 (34, 52), *D. squalens* shows only weak induction in wild-type and none in *Dsace3*^MUTA^, consistent with limited lactose sensing. A possible explanation is transporter specificity; e.g., the highly expressed gene encoding Dicsqu464_2_3103150 may act as a Crt1-like transceptor with reduced affinity or signaling toward lactose.

The role of *Ds*Ace3 in CCR remains unclear. *Dscre1* was induced on cellulose in the wild-type, yet CAZymes were not repressed, suggesting CCR is overridden under these conditions. Glucose represses *Dsace3* expression (47), and in *T. reesei*, *Trace3* overexpression or C-terminal truncation bypasses CCR and enhances cellulase production (33, 34); whether similar *Ds*Ace3 modulation would benefit *D. squalens* remains to be tested.

Our study demonstrates that *Ds*Ace3 is a central regulator of plant biomass degradation in *D. squalens*. Like its characterized orthologs, *Ds*Ace3 governs cellulase/xylanase and ST gene expression and cellulose metabolism. However, notable differences in basidiomycetes, including the absence of other key ascomycete TF orthologs, indicate that Ace3/Roc1 TFs have evolved distinct yet functionally conserved roles in fungal cellulose degradation. Furthermore, direct binding of *Ds*Ace3 to CAZyme promoters, as well as its direct regulatory targets, is yet to be demonstrated. Our study extends prior work on cellulase regulation in basidiomycetes by characterizing the Ace3 ortholog from a wood-degrading white-rot fungus with strong ligninolytic capacity and establishes a framework for future molecular and regulatory analysis required to fully elucidate the function of Ace3/Roc1 in basidiomycete transcriptional networks.

## MATERIALS AND METHODS

### Strains and culture conditions

The *D. squalens* wild-type monokaryotic strain CBS 464.89 (CBS Collection, Westerdijk Fungal Biodiversity Institute, Utrecht, the Netherlands) (55) and *Dsace3* mutant strains (this study) were maintained on 2% MEA at 4°C. For pre-cultivation, 0.5 cm mycelium-covered plugs from MEA were transferred to low-nitrogen asparagine-succinate (LN-AS) agar (56) supplemented with 0.05% glycerol. All experiments were conducted in biological triplicates.

For growth profiling, CBS 464.89 and *Dsace3* mutant strains were cultivated on 2% MEA or LN-AS agar supplemented with 25 mM of D-glucose (Sigma), cellobiose (Fluka), D-galactose (Sigma), lactose (Sigma), D-xylose (Sigma), L-arabinose (Thermo Scientific), or D-mannose (Thermo Scientific), or 1% crystalline cellulose (Fluka), beechwood xylan (Carl Roth), guar gum (Sigma), or pectin (from apple, Sigma), or 3% birch or spruce sawdust as the sole carbon source. Cultures were incubated at 28°C for 3–5 days. To facilitate visualization of fungal growth, cellulose medium was supplemented with 0.002% bromophenol blue sodium salt (Sigma).

For carbon source induction, both strains were cultivated on polycarbonate track-etched membranes (GVS, Healthcare & Life Sciences) placed on MEA at 28°C for 3 days. For HPLC analysis of the extracellular sugars, membranes with mycelium were transferred to a petri dish containing 25 mL of LN-AS broth supplemented with 1% crystalline cellulose (Fluka) for two additional days. For RNA extraction, mycelium was transferred to LN-AS agar plates supplemented with 25 mM of D-glucose (Sigma) or lactose (Sigma), or 1% crystalline cellulose (Fluka) or beechwood xylan (Carl Roth). Additionally, both strains were cultivated on cellobiose (Fluka) for 5 days.

For analysis of secreted proteins and cellulase activity, 50 agar plugs (⌀ 0.5 cm) were collected from 5-day MEA pre-cultures of both strains. Plugs were homogenized in a Waring blender for 5 s with 150 mL of 2% malt extract broth, filtered through autoclaved Miracloth (Calbiochem), and centrifuged (10 min, 4°C, 805 × *g*). The supernatant was discarded, and pellets were resuspended in 20 mL of LN-AS broth. Cultures were inoculated with 2.5 mL of the prepared mycelial suspension in 50 mL of LN-AS broth supplemented with 1% crystalline cellulose (Fluka) and incubated in baffled flasks at 28°C, 120 rpm, for 19 days.

### Genomic DNA extraction, sequencing, and annotation

High molecular weight DNA was extracted from mycelium grown on MEA using the protocol of Doyle and Doyle (57) with minor modifications. Flash-frozen biomass was ground to a fine powder in a frozen mortar with liquid nitrogen and incubated in 2% CTAB buffer containing proteinase K, PVP-40, and β-mercaptoethanol for 30 min to 1 h at 50°C. After centrifugation, the supernatant was transferred to a new tube and treated with 200 µL of 50 mM phenylmethanesulfonyl fluoride for 10 min at room temperature. After two rounds of extraction with 24:1 chloroform:isoamyl alcohol, the upper phase was transferred to a new tube, and 1/10 vol 3 M sodium acetate was added and gently mixed. DNA was precipitated with isopropanol, collected by centrifugation, washed with 70% ethanol, air-dried for 5–10 min, and dissolved thoroughly in elution buffer at room temperature followed by RNAse treatment. DNA purity was measured with Nanodrop; DNA concentration was measured with Qubit HS kit (Invitrogen); and DNA size was validated by Femto Pulse System (Agilent).

An input of 1,500 ng of genomic DNA was sheared to approximately 10 kb fragments using the g-TUBE (Covaris). The sheared DNA was processed with exonuclease to remove single-stranded ends, followed by treatment with DNA damage repair enzyme mix, end-repair/A-tailing mix, and ligation with barcoded overhang adapters from SMRTbell Barcoded Adapter Plate 3.0 (PacBio) using the SMRTbell Express Template Prep Kit 2.0 (PacBio). Up to 16 libraries were pooled at equimolar concentrations and purified with AMPure PB Beads (PacBio). Pooled libraries were size-selected using the 0.75% agarose gel cassettes with Marker S1 and High Pass protocol on the BluePippin (Sage Science). PacBio Sequencing primer was then annealed to the SMRTbell template library, and sequencing polymerase was bound using the Sequel II Binding kit 2.0. The prepared SMRTbell template libraries were sequenced on a Sequel IIe sequencer (PacBio), using TBD sample-dependent sequencing primer, 8M v.1 SMRT cells, and v.2.0 sequencing chemistry with 1 × 1,800 sequencing movie run times.

Subreads were filtered to remove sequencing artifacts. Mitochondrial genomes were assembled separately with the circular consensus sequencing (CCS) reads using an in-house tool (assemblemito.py), used to filter the CCS reads. The resulting assemblies were polished with two rounds of RACON (v.1.4.13) with the parameters [-u -t 36] (https://github.com/lbcb-sci/racon). The mitochondria-filtered CCS reads were then assembled with Hifiasm (v.0.15.4) with the options [-t 32—primary] (58). The resulting assemblies were further refined by two additional rounds of RACON polishing with the same parameters.

The Phase Genomics Proximo Hi-C (Fungal) Kit Protocol (v.4.0) was used to process approximately 500 mg of *D. squalens* CBS 464.89 fungal tissue. The prepared libraries were quantified using KAPA Biosystems’ next-generation sequencing library qPCR kit and run on a Roche LightCycler 480 real-time PCR instrument. Sequencing of the flow cell was performed on the Illumina NovaSeq sequencer using NovaSeq XP (v.1.5) reagent kits and an S4 flow cell, following a 2 × 151 indexed run recipe.

For Hi-C improvements, restriction cut sites were generated using generate_site_positions.py from Juicer (v.1.6) (59–61) with DpnII as the restriction enzyme. A contact map was then generated using Juicer.sh (v.1.6) and 1.625 Gbp of QC filtered data from Hi-C library. Manual breaks and joins were made using Juicebox (v.1.11.08).

The genome was annotated using the JGI Annotation pipeline (62) and both Illumina RNA-Seq and PacBio Iso-Seq processed reads. The *D. squalens* CBS 464.89 genome v.2.0 can be accessed at https://mycocosm.jgi.doe.gov/Dicsqu464_2/.home.html.

### RNA isolation and sequencing

For Iso-Seq RNA sequencing, 500 ng of total RNA was used as input. Full-length cDNA synthesis was performed using NEBNext Single Cell/Low Input cDNA Synthesis & Amplification Module kit. First-strand cDNA was amplified using NEBNext High-Fidelity 2× PCR Master Mix with barcoded cDNA PCR primers and 11–14 cycles of amplification. The amplified cDNA was purified using 1.0× AMPure PB beads ratio for non-size-selected libraries. For size-selected libraries, cDNA was purified using a 0.4× AMPure PB bead-to-sample ratio to select for fragments >1 kb or 0.45× ratio for fragments >700 bp.

cDNA of similar sizes was pooled at equimolar ratios in a designated degree-of-pool using the PacBio Multiplexing Calculator. The pooled samples are end-repaired, A-tailed, and ligated with overhang non-barcoded adaptors using the SMRTbell Express 2.0 kit. Annealing of the PacBio Sequencing primer, binding of the polymerase, and sequencing of the resulting cDNA libraries were performed as described for the genomic DNA.

For RNA-seq for genome annotation, library preparation and sequencing were performed at the Joint Genome Institute (JGI, Walnut Creek, USA) as previously described (22) with two minor changes. First, plate-based RNA sample prep was performed on the Hamilton Vantagerobotic liquid handling systems. Second, sequencing of the flow cell was performed on the Illumina NovaSeq sequencer using NovaSeq XP (v.1.5) reagent kits, 10B flow cell, following a 2 × 151 indexed run recipe.

For RNA-seq of cultivations induced by carbon sources, fungal mycelia were harvested from 1.5 cm of the colony periphery, and total RNA was extracted using a TRIzol-based method as described earlier (63). RNA quantity and purity were assessed using a NanoDrop One spectrophotometer (Thermo Scientific), while integrity was evaluated using an Agilent 2100 Bioanalyzer with the RNA 6000 Nano Kit (Agilent Technologies). RNA-seq was performed as described above. Reads were mapped to the *D. squalens* CBS 464.89 genome v.2.0.

### Identification of orthologs and sequence conservation analysis

A total of 44 characterized Zn(II)2Cys6 TFs involved in plant biomass utilization in ascomycetes and basidiomycetes were selected based on a literature review (Table S4). Genome-wide ortholog cluster analysis was conducted on 35 fungal species, including the 18 ascomycetes and one basidiomycete where these TFs were originally characterized, as well as *D. squalens* and 15 additional basidiomycetes with high-quality genome assemblies (Table S3).

Proteomes of the selected species were downloaded from JGI MycoCosm (64), except for *Talaromyces pinophilus*, which was obtained from UniProt (https://www.uniprot.org/). Ortholog groups were identified using OrthoFinder (v.2.5.4) (65) with default parameters.

For domain validation, InterProScan (v.5.51-85.0) (66) was used to annotate proteins within orthogroups containing at least one characterized TF. Proteins with domain architectures matching those of the characterized TFs were selected for phylogenetic analysis. Protein sequences were manually curated to remove paralogs and incorrect gene models.

Phylogenetic reconstruction followed a pipeline previously described (46). As an outgroup, four FacB protein sequences from Aspergilli were selected (67). Amino acid sequences of Ace3/Roc1 and AmyR TFs were aligned using Clustal Omega (https://www.ebi.ac.uk/jdispatcher/msa/clustalo) to evaluate sequence similarities. Total and grouped per-residue conservation scores were calculated and smoothed (*k* = 20) on Jalview (v.2.11.4.1) using the algorithm from Livingstone and Barton (68). The specific residue conservation in the alignment was visualized using ESPript 3.0 (69).

### Construction of the *Dsace3* mutant strains

The *Dsace3* gene disruption was designed based on the *D. squalens* CBS 464.89 v.1.0 genome available at https://mycocosm.jgi.doe.gov/Dicsqu464_1.home.html. Notably, the *Dsace3* locus is identical in v.2.0. Geneious Prime (v.11.1.4) was used to identify and evaluate potential CRISPR target sites within the *Dsace3* locus, assessing both on-target activity (70) and off-target specificity scores (71).

Guide RNAs (gRNAs) were synthesized using the GeneArt Precision gRNA Synthesis Kit (Thermo Scientific) and custom primers (Table S6), following the manufacturer’s instructions. Ribonucleoprotein (RNP) complexes were assembled with Cas9-NLS (Thermo Scientific) as described in reference 43. To introduce two nonsense mutations and a silent mutation disrupting a BamHI restriction site, a custom 120 nt single-stranded oligodeoxynucleotide (ssODN) was designed as a homology-directed repair donor and synthesized by Eurofins Genomics (Konstanz, Germany) (Fig. S3; Table S6).

For gene disruption, polyethylene glycol-mediated co-transformation of RNP complexes and ssODNs targeting the *Dsace3* and *sdi1* loci was performed as previously described (43, 72). *sdi1* transformants were selected on regeneration agar supplemented with 0.5–2.0 µg/mL carboxin (Sigma-Aldrich). Successful *Dsace3* disruption was verified by genomic DNA extraction (73) and PCR amplification using custom primers (Table S6). PCR products were screened by BamHI (FastDigest, Thermo Scientific) restriction enzyme assay and visualized on a 1% agarose gel. Transformants lacking the restriction site were further confirmed by Sanger sequencing (Macrogen, Amsterdam, The Netherlands) (Fig. S3).

### Analysis of extracellular sugars and proteins

Liquid culture samples were collected after 5 days (petri dish cultures) or 5, 10, 15, and 19 days (baffled flask cultures) of incubation. Soluble and insoluble fractions were separated by centrifugation at 16,000 × *g* for 10 min.

The supernatant from petri dish cultures was analyzed for extracellular sugars using HPAEC-PAD, following a previously described protocol (74). The supernatant from baffled flask samples was analyzed for extracellular proteins on Mini-PROTEAN TGX Stain-Free 10% SDS-PAGE gels (Bio-Rad) with PageRuler Plus Prestained Protein Ladder (Thermo Scientific). Enzymatic activities of BGLs, CBHs, and EGLs in the supernatant were measured as previously described (75).

### Transcriptome data analysis

Gene expression levels and differential expression analysis were performed using DESeq2 (v.1.30.1) (76, 77) and pheatmap (v.1.0.12), implemented in R (v.4.3.0), following a previously established pipeline (28). Predicted gene annotations for CAZymes, TFs, STs, and sugar metabolic genes were retrieved from *D. squalens* CBS 464.89 genome v.2.0 and manually curated on prior studies (22, 28, 45, 46, 63). To identify putative extracellular peptidases, predicted peptidases were retrieved from *D. squalens* CBS 464.89 genome v.2.0 and their protein sequences were analyzed using SignalP 6.0 (78). *D. squalens* predicted orthologs of *S. cerevisiae* Sec61 complex members Sec61 (Sacce1_4429), Sbh1 (Sacce1_2035), and Ss1 (Sacce1_1109) were identified using protein BLAST against the *D. squalens* CBS 464.89 genome v.2.0.

## Data Availability

The *D. squalens* CBS 464.89 genome (v.2.0) genome assembly and annotations are available at MycoCosm (http://jgi.doe.gov/fungi). The PacBio and Illumina data are available in GenBank under BioProject no. PRJNA1080834. The read counts for each sample of RNA-seq were deposited in NCBI’s Sequence Read Archive under BioProject accession nos. PRJNA1362433, PRJNA1362442, PRJNA1362443, PRJNA1362457, PRJNA1362461, PRJNA1362464, PRJNA1362465, PRJNA1362466, PRJNA1362471, PRJNA1362475, PRJNA1362476, PRJNA1362496, PRJNA1362503, PRJNA1362510, PRJNA1362512, PRJNA1362513, PRJNA1362526, PRJNA1362530, PRJNA1362538, PRJNA1362541, PRJNA1362544, PRJNA1362570, PRJNA1362573, PRJNA1362605, PRJNA1362607, PRJNA1362610, PRJNA1362611, PRJNA1362647, PRJNA1362653, and PRJNA1362692.

## SUPPLEMENTAL MATERIAL

Supplemental figures aem.01548-25-s0001.pdf Figures S1 to S5.

Table S1 aem.01548-25-s0002.xlsx Comparative assembly and annotation statistics for v1.0 and v2.0 of *Dichomitus squalens* CBS 464.89.

Table S2 aem.01548-25-s0003.xlsx Curated gene lists of predicted carbohydrate-active enzyme (CAZy), transcription factor (TF), sugar transporter (ST), and sugar metabolic genes in *Dichomitus squalens* CBS 464.89 v2.0 annotated genome at JGI MycoCosm database.

Table S3 aem.01548-25-s0004.xlsx Taxonomy, strain ID, and source information for 18 ascomycete and 17 basidiomycete proteomes used in orthology and phylogeny.

Table S4 aem.01548-25-s0005.xlsx List of selected Zn(II)2Cys6 fungal transcription factors (TFs) controlling plant biomass modifying enzymes in filamentous fungi.

Table S5 aem.01548-25-s0006.xlsx Gene expression levels on plant biomass-derived mono-, short oligo-, and polysaccharides, and lactose cultivations of *Dichomitus squalens* CBS 464.89 and *Dsace3*^MUTA^ compared to D-glucose.

Table S6 aem.01548-25-s0007.xlsx Custom oligos used for the disruption of *Dichomitus squalens ace3* and genetic analyses.

ASM does not own the copyrights to Supplemental Material that may be linked to, or accessed through, an article. The authors have granted ASM a non-exclusive, world-wide license to publish the Supplemental Material files. Please contact the corresponding author directly for reuse.
