# Supplementary material for: The Zn(II)2Cys6 transcription factor DsAce3 is a major activator of cellulases in the white-rot fungus Dichomitus squalens
Source: Appl Environ Microbiol. 2026 Feb 9;92(3):e01548-25. doi: 10.1128/aem.01548-25 (PMC12997857; doi:10.1128/aem.01548-25)
Supplement: Supplemental figures — Figures S1 to S5. [file aem.01548-25-s0001.pdf]

The Zn(II)2 Cys6 transcription factor *Ds*Ace3 is a major activator of cellulases in the white-rot fungus *Dichomitus squalens*

Supplementary figures

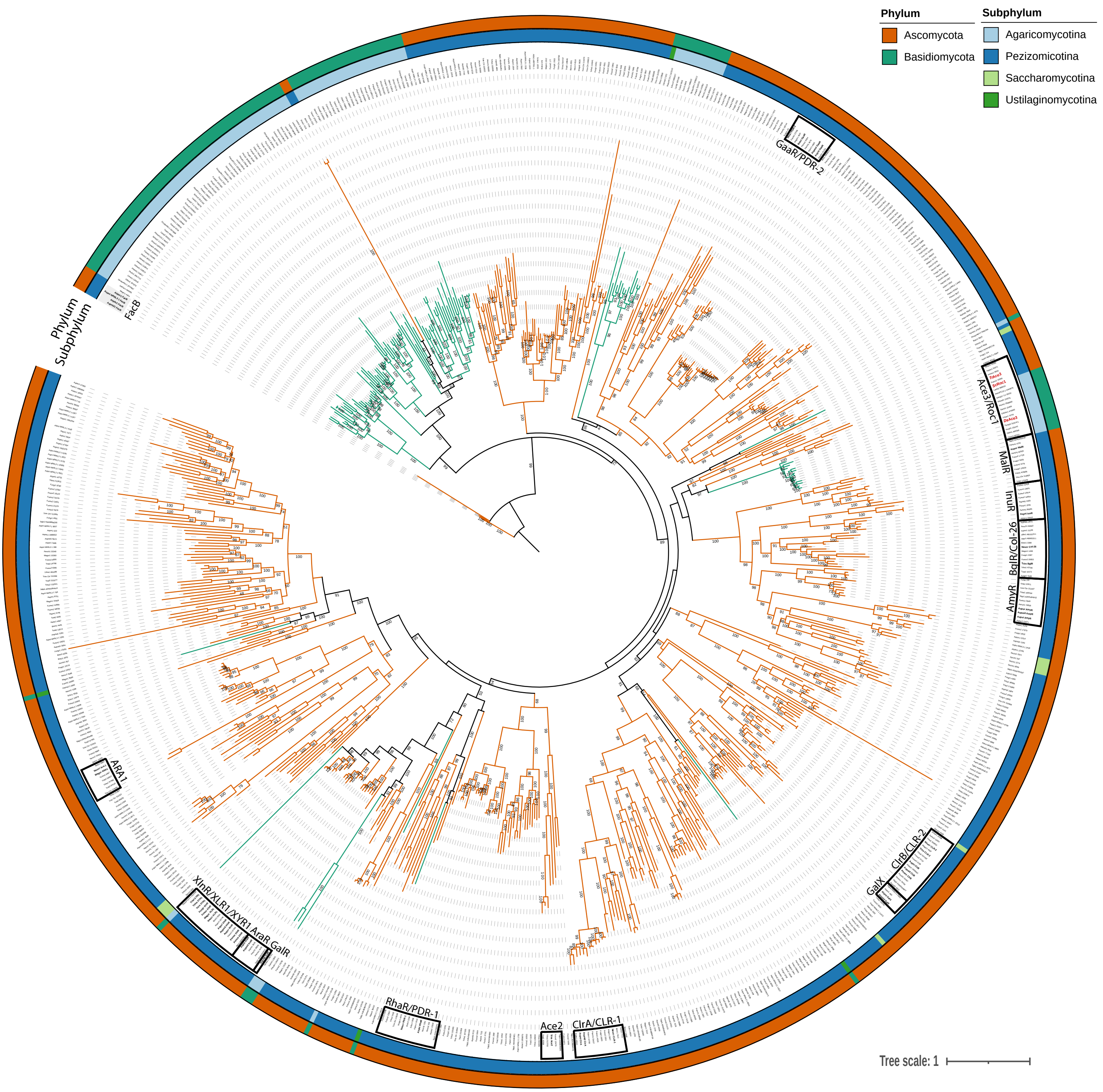

Figure S1. Maximum-likelihood phylogenetic tree of selected Zn(II)2Cys6 fungal transcription factors involved in the regulation of plant biomass-modifying enzymes and their predicted orthologs from Ascomycota (orange) and Basidiomycota (green). Clades corresponding to distinct transcription factor groups (e.g. Ace3/Roc1, XlnR/XYR1, and AmyR) are indicated. Protein IDs from the source proteome and species abbreviations (as listed in Table S3) are shown. Experimentally characterized transcription factors are indicated in bold. *Dichomitus squalens Ds*Ace3 and its characterized orthologs are highlighted in red and bold. Bootstrap support values ≥ 50 are shown at branch points.

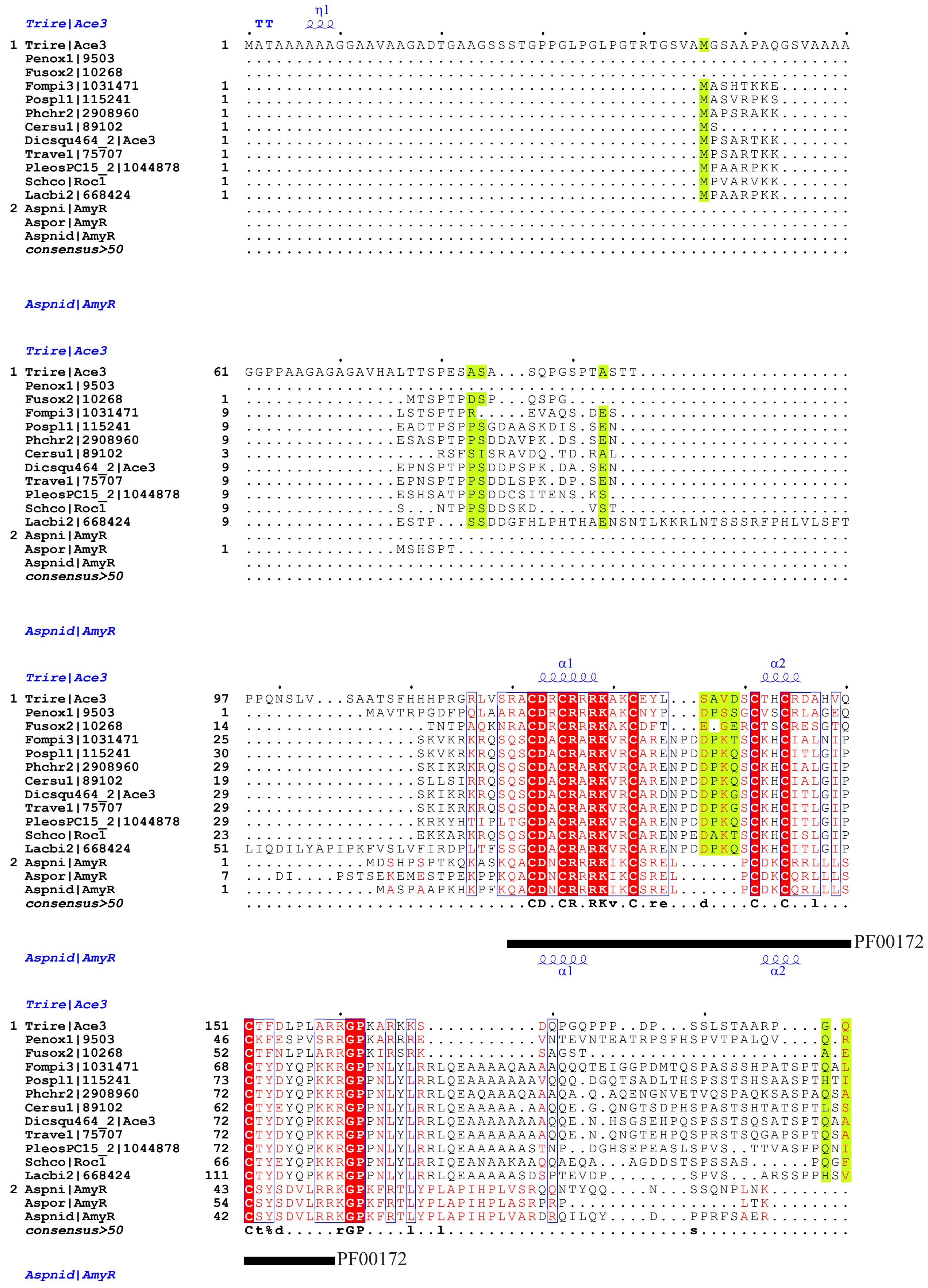

Figure S2. ClustalW-aligned (https://www.ebi.ac.uk/jdispatcher/msa/clustalo) protein sequences of selected ascomycete and basidiomycete Ace3/Roc1 orthologs and characterized AmyR transcription factors. Protein IDs from the source proteome and species abbreviations (as listed in Table S3) are shown. Hydrophobic and flexibility-related residues conserved in Ace3/Roc1 are indicated by red and brown triangles, respectively. Predicted PFAM domains are indicated with black rectangles. *Trichoderma reesei* Ace3 and *Aspergillus nidulans* AmyR protein models were obtained from AlphaFold Protein Structure Database (https://alphafold.com/; AF-A0A5C1J077-F1-v4, AF-Q9Y728-F1-v4). Consensus sequence of Ace3/Roc1 and AmyR is shown. The alignment was visualized using ESPRIPT3.0.

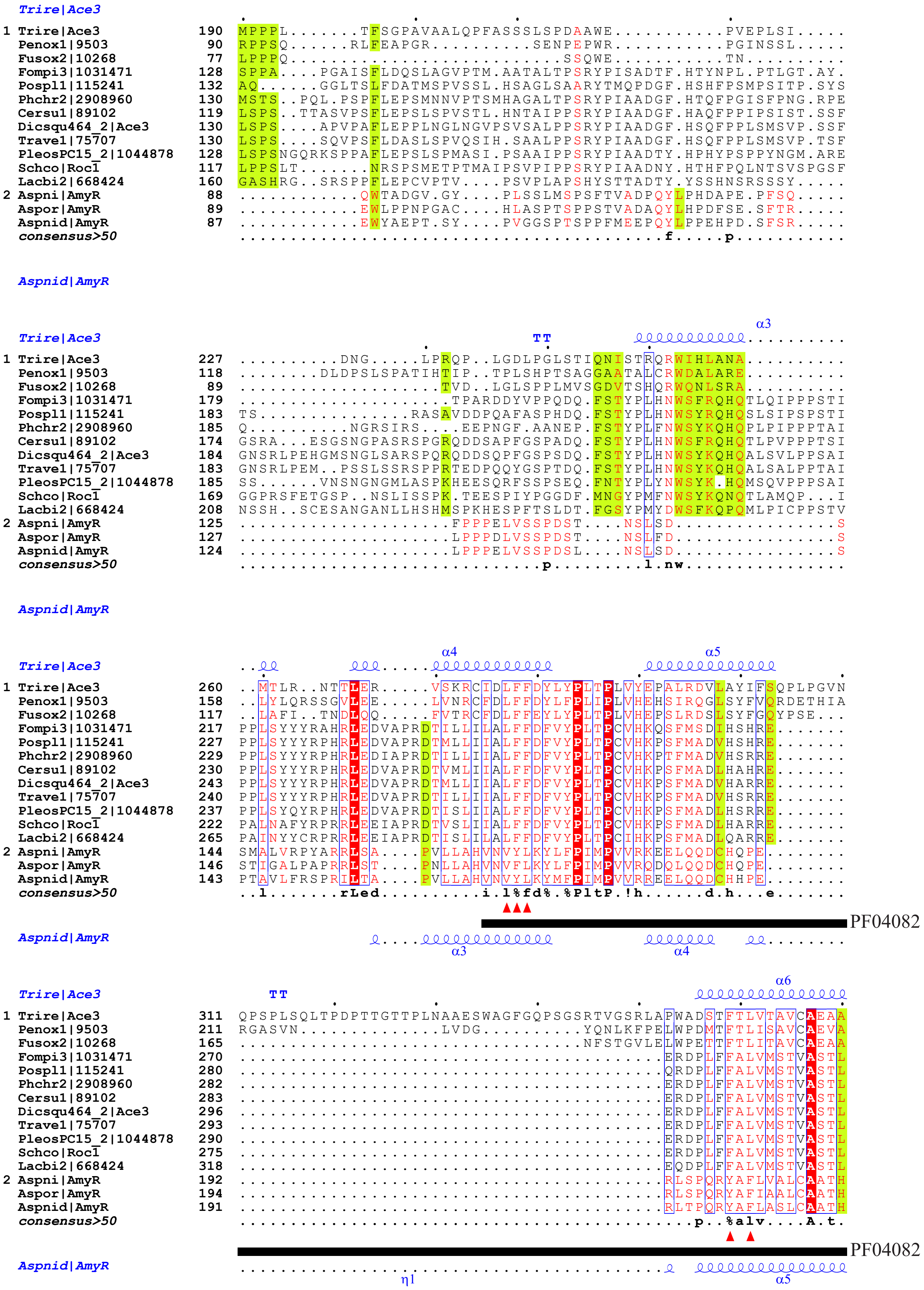

Figure S2. ClustalW-aligned (https://www.ebi.ac.uk/jdispatcher/msa/clustalo) protein sequences of selected ascomycete and basidiomycete Ace3/Roc1 orthologs and characterized AmyR transcription factors. (Cont.)

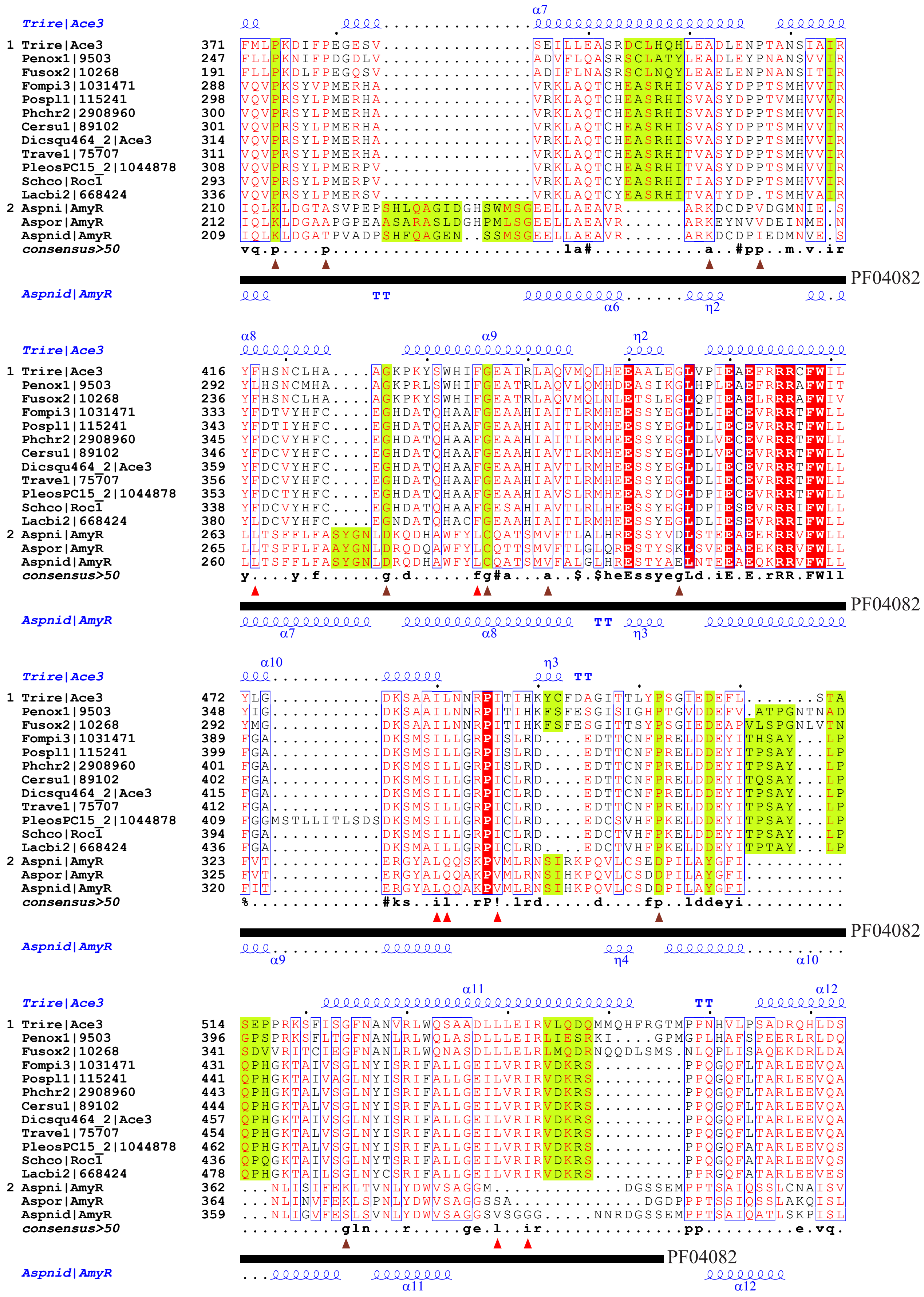

Figure S2. ClustalW-aligned (https://www.ebi.ac.uk/jdispatcher/msa/clustalo) protein sequences of selected ascomycete and basidiomycete Ace3/Roc1 orthologs and characterized AmyR transcription factors. (Cont.)

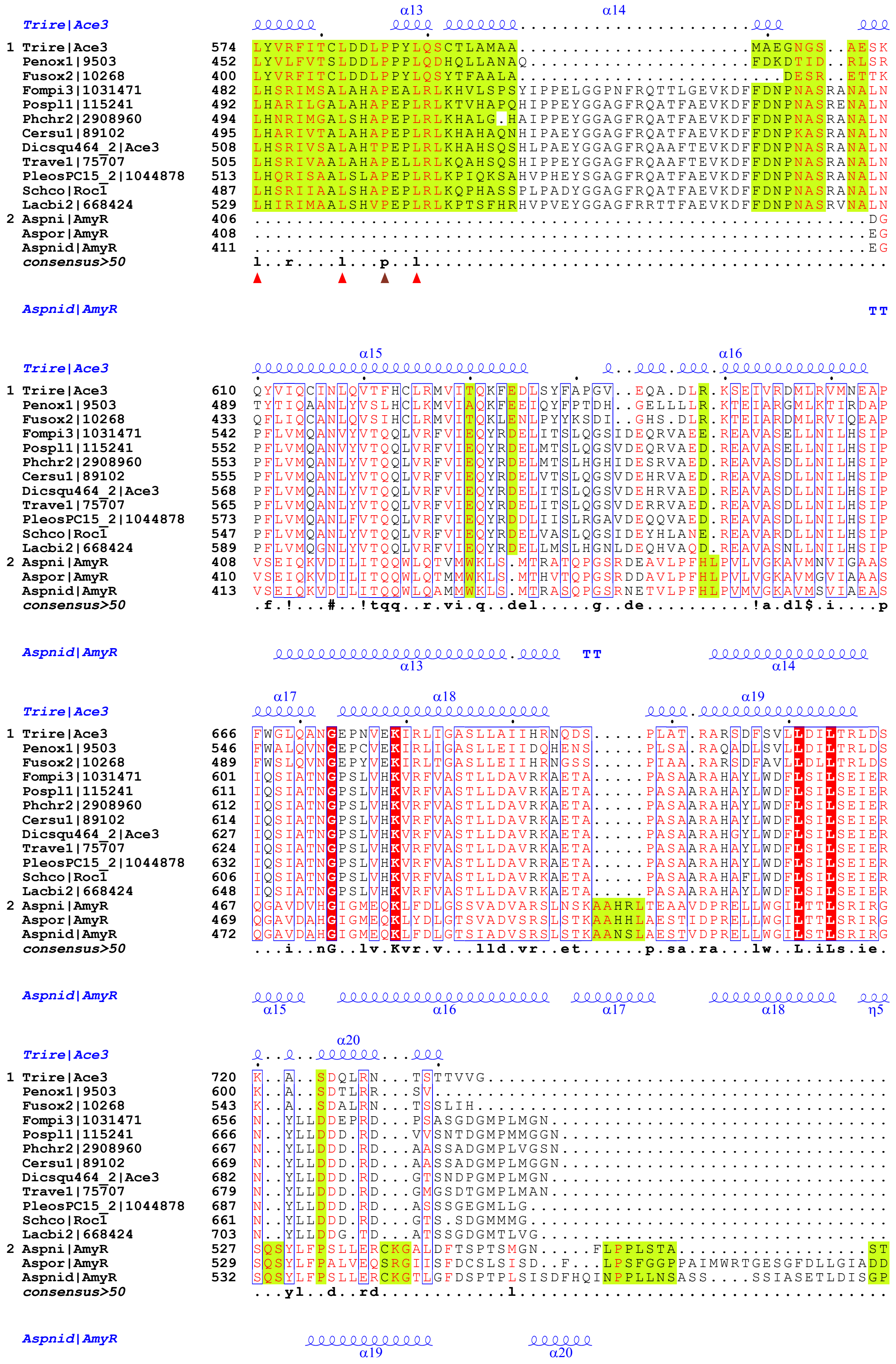

Figure S2. ClustalW-aligned (https://www.ebi.ac.uk/jdispatcher/msa/clustalo) protein sequences of selected ascomycete and basidiomycete Ace3/Roc1 orthologs and characterized AmyR transcription factors. (Cont.)

```
   Trire|Ace3

 1 Trire|Ace3                 ............................................................
   Penox1|9503                ............................................................
   Fusox2|10268               ............................................................
   Fompi3|1031471             ............................................................
   Pospl1|115241              ............................................................
   Phchr2|2908960             ............................................................
   Cersu1|89102               ............................................................
   Dicsqu464_2|Ace3           ............................................................
   Trave1|75707               ............................................................
   PleosPC15_2|1044878        ............................................................
   Schco|Roc1                 ............................................................
   Lacbi2|668424              ............................................................
 2 Aspni|AmyR            564  WEE...ETGLAVVSVPENP.........................................
   Aspor|AmyR            584  LQERENEGGEGIVV.A..............G.............................
   Aspnid|AmyR           588  WPA...ADDNTMDSSTGSRTTNSCAWEITSGDTNDQNQTSDQPPSLSGSSSVPLSVSRMH
   consensus>50               ............................................................

   Aspnid|AmyR


   Trire|Ace3

 1 Trire|Ace3                 ..................
   Penox1|9503                ..................
   Fusox2|10268               ..................
   Fompi3|1031471             ..................
   Pospl1|115241              ..................
   Phchr2|2908960             ..................
   Cersu1|89102               ..................
   Dicsqu464_2|Ace3           ..................
   Trave1|75707               ..................
   PleosPC15_2|1044878        ..................
   Schco|Roc1                 ..................
   Lacbi2|668424              ..................
 2 Aspni|AmyR                 ..................
   Aspor|AmyR            600  ......EEISF.......
   Aspnid|AmyR           645  SSTSMAAELSFQAGRLLT
   consensus>50               ..................

   Aspnid|AmyR                    α21
```

Figure S2. ClustalW-aligned (https://www.ebi.ac.uk/jdispatcher/msa/clustalo) protein sequences of selected ascomycete and basidiomycete Ace3/Roc1 orthologs and characterized AmyR transcription factors. (Cont.)

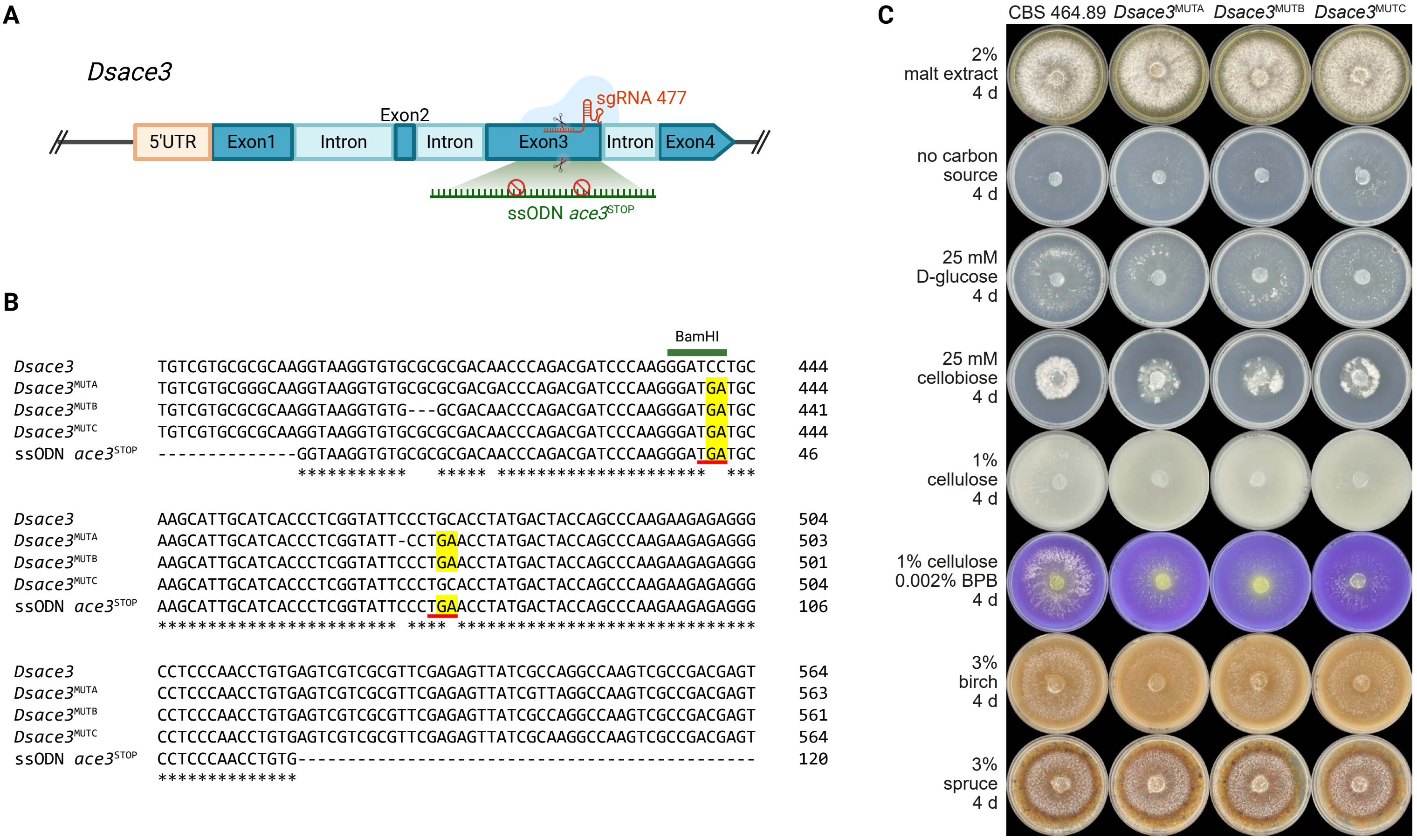

Figure S3. CRISPR/Cas9-mediated gene disrruption of *Dsace3* in *Dichomitus squalens*. A) Schematic representation of two nonsense mutations introduced by Cas9-mediated homologous recombination at the *Dsace3* locus. B) Sanger sequencing of the *Dsace3* locus from *Dsace3* mutant strains compared to the wild-type and the single-stranded oligodeoxynucleotide (ssODN) homology donor. Nonsense mutated codons are underlined in red and the corresponding point mutations are highlighted in yellow. The introduced BamHI cutting site is indicated in green. C) Growth profiling of the wild-type strain CBS 464.89 and *Dsace3* mutant strains on malt extract, no carbon source, plant biomass-derived mono-, di-, and polysaccharides, and wood substrates. BPB: bromophenol blue.

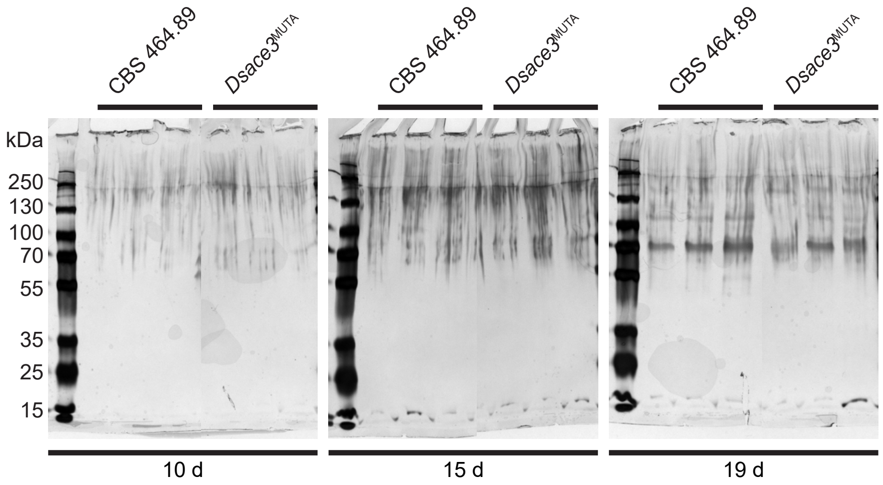

Figure S4. SDS-PAGE analysis of the extracellular proteins from biological triplicate shaken liquid cultivations of the wild-type strain CBS 464.89 and *Dsace3*^{MUTA} strain grown on 25 mM lactose.

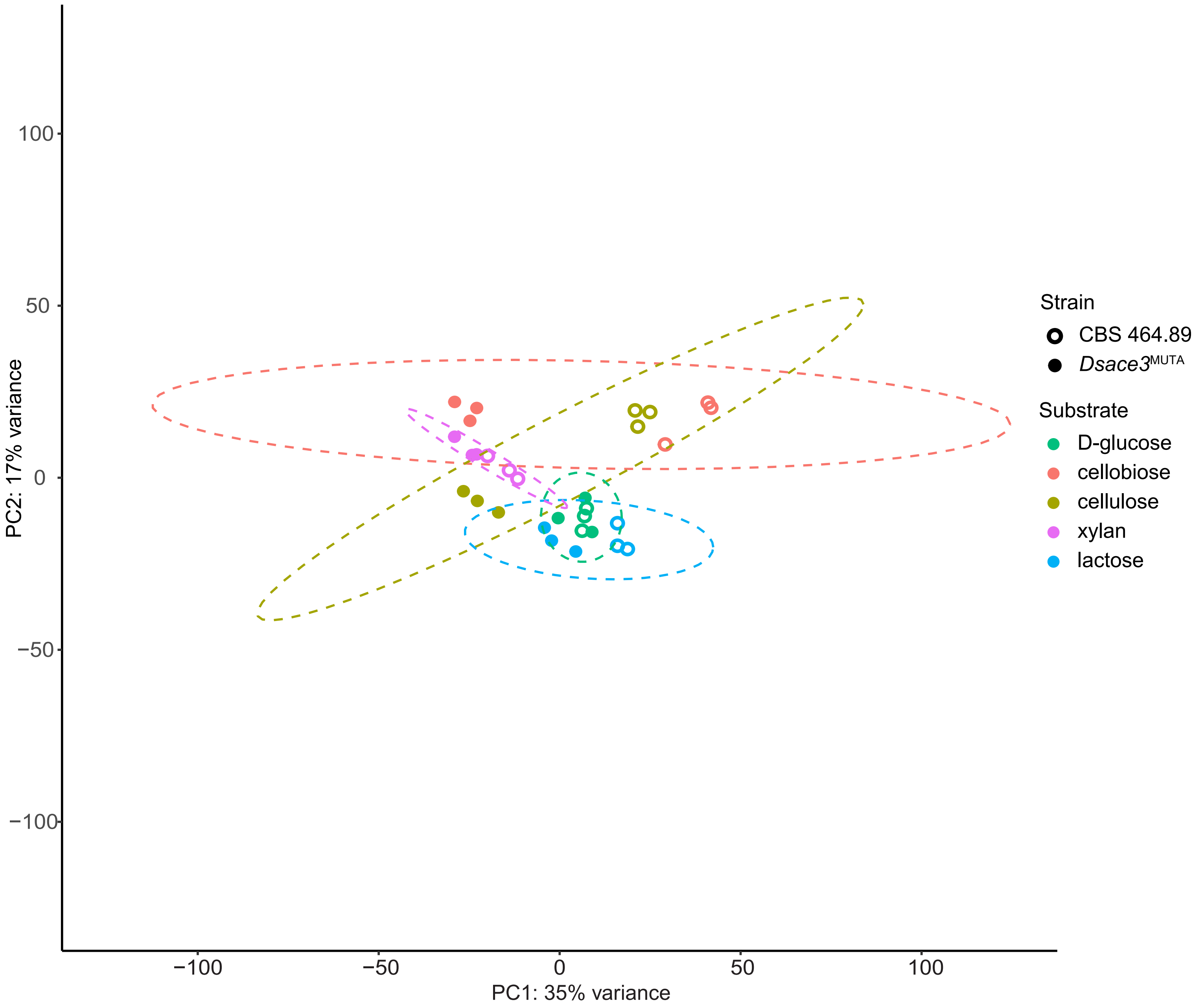

Figure S5. PCA of the transcriptomes from *Dichomitus squalens* CBS 464.89 and *Dsace3*^{MUTA} grown on plant biomass-derived mono-, short oligo- and polysaccharides and lactose grouped by substrate. Ellipses correspond to 0.05 confidence intervals. The variance-stabilized transformed counts of 4871 genes with DESeq2 normalized counts ≥10 in at least one tested condition were analyzed.
